# The role of dietary diversity in the response to treatment of uncomplicated severe acute malnutrition among children in Niger: a prospective study

**DOI:** 10.1186/s40795-018-0242-y

**Published:** 2018-09-20

**Authors:** Isabel Madzorera, Christopher Duggan, Fatou Berthé, Rebecca F. Grais, Sheila Isanaka

**Affiliations:** 1 Department of Nutrition, Harvard TH Chan School of Public Health, 677 Huntington Ave, Boston, MA 02115 USA; 2 Division of Gastroenterology and Nutrition, Children’s Hospital Boston, 300 Longwood Avenue, Boston, MA 02115 USA; 3 Epicentre Niger, Maradi, Niger; 4 Department of Research, Epicentre, 8 rue Saint Sabin, 75011 Paris, France; 5 Department of Global Health and Population, Harvard TH Chan School of Public Health, 677 Huntington Ave, Boston, MA 02115 USA

**Keywords:** Severe acute malnutrition, Nutritional status, Nutritional recovery, Dietary diversity, Food groups, Niger

## Abstract

**Background:**

Community-based treatment of severe acute malnutrition (SAM) has proven to be safe and cost-effective, although identifying additional factors that can increase recovery and decrease treatment failure may improve program effectiveness. We examine the association of dietary diversity and clinical and program treatment outcomes among children treated for uncomplicated SAM in Niger.

**Methods:**

Two thousand four hundred twelve children were enrolled in a randomized trial of routine amoxicillin in the treatment of uncomplicated SAM from 2012 to 2014. All children received ready to use therapeutic food (RUTF) and standard clinical care. Child dietary diversity was assessed using a 7-day food frequency questionnaire and 8-food group diet diversity score. We assessed the association of dietary diversity at admission with nutritional recovery, hospitalization, and death at program discharge and 12 weeks, and weight and height gain.

**Results:**

Food groups most commonly consumed by children in seven days preceding SAM treatment were cereals, roots and tubers (*N* = 2364, 99.5%) and vitamin A rich fruits and vegetables (*N* = 2253, 94.8%). Egg (*N* = 472, 19.9%) and dairy (*N* = 659, 27.7%) consumption was low. Mean (SD) diet diversity score was significantly lower in the lean vs. non-lean season [2.7 (1.1) vs. 2.9 (1.0)]. There was no evidence that dietary diversity increased nutritional recovery at discharge (RR: 1.02, 95% CI: 1.00, 1.04) or 12 weeks (RR: 0.98, 95%CI: 0.94, 1.02). No significant association was found with risk of hospitalization or death, or weight and height gain. Egg consumption was protective against death at discharge (RR: 0.53, 95% CI: 0.39, 0.70) and 12 weeks (RR: 0.66, 95% CI: 0.45, 0.96). Vitamin A rich fruits and vegetable consumption was associated with greater risk of mortality in children at discharge (RR: 1.30, 95% CI: 1.08, 1.56) and 12 weeks (RR: 1.19, 95% CI: 1.03, 1.36).

**Conclusions:**

We did not find evidence that dietary diversity influenced nutrition recovery or response to treatment for children with uncomplicated SAM in Niger. It is feasible consumption of nutrient-dense foods like eggs may be important for recovery from SAM. There is need for continued research to further elucidate drivers of nutritional recovery from acute malnutrition in different settings.

**Trial registration:**

Trial registration number: ClinicalTrials.gov NCT01613547. Registered May 26, 2012.

## Background

Nearly 17 million children under 5 years of age worldwide, and 4.1 million in Africa, are affected by severe acute malnutrition (SAM) [1–3]. Approximately 7% of all deaths in children under 5 years can be attributed to SAM and children with weight-for-height Z (WHZ) score < − 3 have a 9-fold higher risk of death compared to healthy children [2]. Since 2006, SAM treatment has focused on outpatient therapy for children with no complications and appetite and reserved inpatient management for children with clinical complications [4, 5]. This community-based approach has proven to be safe and cost-effective, though treatment failure, default and mortality can reduce the potential effectiveness of programs and limit their scale-up [6–9].

Understanding the factors that drive nutritional recovery during SAM treatment could be used to improve program success. One measure of diet quality, dietary diversity (DD), has been associated with a reduced risk of wasting and stunting, as well as increased micronutrient adequacy and caloric sufficiency among children [10–13]. Dietary diversity, among other factors, can contribute to the risk of acute wasting, but its role in SAM recovery and the response to treatment is not well understood [9, 14, 15].

We hypothesized dietary diversity at admission, as a marker of micronutrient adequacy, may enhance a child’s ability to recover from nutritional deficit by increasing micronutrient availability for recovery and repair processes and enhancing immune function, and promote nutritional recovery. Further, diversified diets may also sustain and maintain recovery from severe acute malnutrition and prevent relapse. We therefore examined the prospective association of dietary intake and dietary diversity measured before treatment for SAM with nutritional recovery, hospitalization, and death at program discharge and 12 weeks and anthropometric outcomes of weight and height gain among children treated for uncomplicated SAM in Niger.

## Methods

### Study setting and population

The study was conducted in the Madarounfa rural health district in south-central Niger, in the Sahel region, a region affected by seasonal fluctuations in food availability, infectious illnesses and high rates of acute malnutrition among young children. The study population included 2412 children enrolled in a randomized trial of routine amoxicillin in the treatment of uncomplicated SAM conducted from 2012 to 2014 (ClinicalTrials.gov Identifier, NCT01613547) [16]. In brief, children were enrolled in the parent trial if they presented to health centers for outpatient treatment of uncomplicated SAM, resided within 15 km of study health centers, were available for 12 weeks of follow up and had no clinical complications requiring antibiotic treatment at admission. Children also could not have been admitted to a nutrition treatment program in the preceding 3 months or have congenital abnormalities. The inclusion criteria for outpatient SAM treatment were age 6 to 59 months, WHZ < − 3 based on World Health Organization (WHO) growth standards, or mid-upper arm circumference (MUAC) < 115 mm. Other inclusion criteria included sufficient appetite for successful oral nutrition and absence of bipedal edema or clinical complications requiring hospitalization [16].

### Study design and interventions

The parent study was a randomized, double-blind placebo-controlled trial. Children were randomized to receive routine amoxicillin or placebo at admission, but otherwise received standard clinical care and ready-to-use therapeutic food for the outpatient treatment of SAM in line with guidelines from Médecins Sans Frontières (MSF) and the Ministry of Health of Niger [16]. Detailed descriptions of the study design and methods are provided elsewhere [16].

### Study procedures and follow-up

At admission, study nurses interviewed caregivers and collected socio-demographic information, including child feeding practices, immunization status, child illnesses and medical history, as well as maternal characteristics and household socioeconomic status. Hemoglobin status (HemoCue Hb 301, HemoCue, Angelholm, Sweden) and malaria infection (SD Bioline Malaria Antigen P.f, Standard Diagnostics Inc., Republic of Korea) were assessed in all children at baseline.

Follow-up was conducted by study staff at health centers on a weekly basis until discharge from the nutritional program (minimum stay 3 weeks for recovery), and at 4, 8 and 12 weeks after enrollment. At each visit, anthropometric measurements were taken. Weight was measured to the nearest 100 g using a hanging Salter scale (≥ 15 kg) or SECA scale (< 15 kg), and length (standing height for children ≥24 months) using a wooden length/height measuring board. MUAC was measured to the nearest 0.1 cm using a non-stretchable MUAC tape. Study physicians conducted a physical exam and completed a medical history that included health events, medical consultations and treatments for children in the last 7 days. When a child missed a scheduled visit, home visits by a study nurse and community health agent were conducted and anthropometry and clinical condition were assessed.

### Dietary diversity

Diet diversity (DD) has been recognized as a tool for evaluating dietary intake and quality for children in low income settings where gold standard tools such as 7 day diet records are not feasible or are limited by cost [17]. While individual foods are diverse and vary by culture, food groups are limited in number and thus are amenable to study and comparison across contexts. Dietary intake and diversity, the primary exposures of interest, were assessed at baseline using a food frequency questionnaire (FFQ) which asked caregivers if the child had eaten a list of 45 foods (in 12 food categories) in the previous 7 days, and if yes, for how many of these 7 days. As proposed by Dewey et al. [18], 8 food groups were created: grains, roots and tubers; legumes and nuts; dairy products; flesh foods; eggs; vitamin A-rich fruits and vegetables; other fruits and vegetables; fats and oils. A diet diversity score (DDS) can be created by awarding one point for each food group consumed over the past 24 h and 0 otherwise, using a 10 g cutoff per food group for the first seven groups, and a 1 g cutoff for fats and oils. In this analysis, which used FFQ not 24-h recall data, a revised scoring algorithm was developed which awarded one point for each food group consumed at least once per day and scaled, partial points for all less frequent consumption (e.g. if eaten on 1 day in the 7-day recall, 1/7 point was awarded). DDS was computed as the sum of food groups consumed. This 8-food group DDS was selected as it has been shown to be predictive of diet quality of complementary foods for children 6–12 months of age as indicated by correlations with mean nutrient density adequacy for selected micronutrients including iron and vitamin A [18]. We also considered other measures of child dietary diversity including the WHO index to assess infant and young child feeding (2007), a 7-food group measure that included grains, roots and tubers; legumes and nuts; dairy products; flesh foods; eggs; vitamin-A rich fruits and vegetables; and other fruits and vegetables [19]. We excluded 22 children from the analysis that were exclusively breastfed at the time of admission.

### Study outcomes

The primary outcomes of the analysis were nutritional recovery, transfer to hospital and death at program discharge and 12 weeks of follow up. Secondary outcomes included change in child weight (g/kg/d) and height (mm/d) from baseline to weeks 1 and 2, program discharge and 12 weeks of follow up, among the children that recovered. Nutritional recovery in the program was determined at 3 weeks or beyond and was defined as WHZ ≥ − 2 on two consecutive visits and MUAC ≥115 mm. Recovered children had no acute complications or edema for a minimum of 7 days and had completed all antibiotic and anti-malaria treatment at program discharge. Default from the program was defined as 3 or more missed weekly visits. Non-response was defined as when a child did not meet criteria for recovery at 8 weeks. Children with weight loss greater than 5% between two consecutive visits, lack of weight gain after 2 weeks or clinical complications requiring inpatient management were transferred to hospital for inpatient management. Death was considered as all-cause mortality.

### Statistical analysis

We evaluated the socio-economic and demographic characteristics of the study children. Chi-squared tests for dichotomous variables and the Wilcoxon rank sum test for continuous variables were used to assess significant differences between levels of dietary diversity (±4 food groups/day). We described intake of individual food groups and the overall DDS, and compared differences between seasons, using chi-squared tests and the Wilcoxon rank sum test.

We calculated DDS at admission, and evaluated the associations between individual food group intake and DDS with the risk of nutritional recovery, transfer to hospital or death at program discharge and 12 weeks. Separate binomial regression models were used for individual food groups or DDS. Among recovered children, we also evaluated associations of food group intake and DDS with weight and height change using linear regression.

We adjusted for amoxicillin/placebo trial regimen, as well as potential confounders assessed at admission and selected based on univariate significance with the outcome at the level of *P* < 0.20. Covariates considered included: child characteristics (age: 6–11, 12–23 vs. 24–59 months), sex: male vs. female), breastfeeding (yes vs. no), child anthropometric status (wasting: none, moderate vs. severe, stunting: none, moderate, severe and MUAC: < 115 vs. ≥115 mm), child health status (anemia: yes vs. no, respiratory rate, and body temperature), presence of morbidity (yes/no: malaria, tachypnea, upper respiratory infection, cough, nasal discharge, diarrhea, vomiting, and ear pain), child receiving ready to use supplementary foods in the past 7 days (yes/no), vaccination status: yes/no (measles, pertussis, tetanus, and polio), previous consultation for child’s health status (yes/no). We also considered household characteristics (number of children < 5 years, household size, wealth index, household food security index (HFIAS score), bednet use(yes/no)), maternal and paternal characteristics (maternal and paternal age, maternal education (none, koranic school, primary school complete, CEG complete), use of health services (yes/no) and season(lean/non-lean). Confounders were measured at admission.

Finally, to more broadly inform the identification of potential boosters of nutritional recovery, we assessed independent predictors of treatment recovery in this setting. Potential predictors identified using logistic regression models with univariate significance at *P* < 0.20 were included in a final multivariate model. All analyses were conducted using SAS version 9.4.

### Ethics

Ethical approval for the parent study was obtained from Comité Consultatif National d’Éthique, Niger and the Comité de Protection des Personnes, Île-de-France XI, Paris. Written informed consent was obtained from the parent or legal guardian of all participating children.

## Results

The analysis population included 2377 children (Table 1). Most children were aged 12–23 months (*n* = 1058, 44.3%) and were partially breastfed at the time of admission (*n* = 1482, 62.0%). More than one-quarter of children presented with anemia (hemoglobin < 8.5 g/dL, *n* = 690, 28.8%) and more than one-half with malaria infection at admission (*n* = 1324, 55.%). A history of recent infectious morbidity was reported in at least one-third of children at admission (31.7% = diarrhea; 55.4% = malaria).

**Table 1:** Baseline demographic, health and anthropometric characteristics of children in SAM outpatient centers, Niger

Characteristic	N (%) or Mean (SD)	Low dietary diversity	High dietary diversity^a^
N	2390	1676(70.1)	714(29.9)
Child characteristics			
Age**			
6–11 months	845 (35.6)	637(38.0)	208(29.1)
12–23 months	1058 (44.3)	705(42.1)	353(49.4)
24–59 months	487 (20.4)	334(19.9)	153(21.4)
Male sex*	1200 (50.1)	816(48.7)	384(53.8)
Breastfeeding status*			
Exclusive breastfeeding	19 (0.8)	12(0.7)	7(1.0)
Partial breastfeeding	1482 (62.0)	1074(64.1)	408(57.1)
None	889 (37.2)	590(35.2)	299(41.9)
Child nutritional status			
Weight for length/height Z-score	−3.1 (0.6)	−3.1(0.6)	− 3.1(0.6)
N (%) < -3SD	1465 (61.3)	1031(61.5)	434(60.8)
Height-for-Age Z-score			
Mean (SD)	−3.0 (1.2)	−3.0(1.2)	− 3.0(1.2)
Z-score below -2SD	1891 (79.1)	1329(79.3)	562(78.7)
Mid upper arm circumference, mm			
Mean (SD)	112 (4.5)	112(4.5)	113(4.4)
MUAC < 115 mm	1861(77.9)	1308(78.0)	553(77.5)
Clinical and medical history			
Vitamin A supplementation in last 6 months	2082 (89.6)	1451(89.1)	631(90.9)
Measles vaccination*	1107 (46.8)	753(45.3)	354(50.4)
Anemia (Haemoglobin less than < 8.5 g/dL)	690 (28.8)	478(28.5)	212(29.7)
Fever (temperature > 38.5 degrees Celsius)	112 (4.7)	76(4.5)	36(5.0)
Rapid diagnostic test positive for malaria*	1324 (55.4)	903(53.9)	421(59.0)
Diarrhoea in the last 24 h	757 (31.7)	530(31.6)	227(31.8)
Vomiting in last 24 h	138 (5.8)	98(5.9)	40(5.6)
Cough in last 24 h	387 (16.2)	277(16.5)	110(15.4)
Household Characteristics			
Household size	7.3 (3.8)	7.1(3.8)	7.4(3.9)
Children < 5 y	1.9 (1.2)	1.9(1.2)	2.0(1.3)
Maternal age, years	26.9 (6.6)	26.9(6.6)	26.9(6.7)
Maternal education < complete primary school*	2334 (97.9)	1639(98.0)	695 (97.6)
Paternal education < complete primary school*	2191 (94.5)	1545(95.3)	646(92.3)
Household Socio-economic status			
Main source of energy for cooking is wood or charcoal	2389(100)	1675(100)	714(100)
Main source of household sanitation is bucket or bush**	1517 (63.5)	1117(66.7)	400(56.0)
Household food security score (HFIAS)^b^			
Lean season (May–August)	6.5 (5.9)	7.4(6.0)	4.0(4.9)
Not lean season (September to April)	5.8 (5.6)	6.4(5.7)	4.6(5.2)

*Significant at 0.05 level; **significant at < 0.001 level

^a^High dietary diversity was defined intake of ≥4 foods groups

^b^HFIAS score based on sum of score from 8 questions [34]. Maximum score is 24

Table 2 shows the consumption of food groups and DDS at admission. Overall diets in the study population showed limited diversity and were based on consumption of staples and plant based foods. Nearly all children consumed cereals (*n* = 2364, 99.5%) and vitamin A rich fruits and vegetables (*n* = 2253, 94.8%) on a daily basis. About half of children ate legumes (*n* = 1582, 66.6%) and meat (*n* = 1199, 50.4%) in the last 7 days, but usually only 2–3 per week. Dairy was not commonly consumed among children (*n* = 659, 27.7%), but when available, it was consumed 4 times per week. Eggs were consumed by one in five children (*n* = 472, 19.9%), and only twice per week in those reporting consumption. In the lean season when household food availability and access are low and the rainy season had begun, there was significantly lower consumption of legumes, flesh foods, fruits and vegetables and fats and oils. Mean (SD) DDS in the lean season was 2.7 (1.1) food groups/day and the non-lean season it was 2.9 (1.0) food groups/day.

**Table 2:** Mean consumption of food groups and DD by season for children with SAM

		Lean season (May–August) *N* = 959		Non-lean season (September–April) *N* = 1418	
Food group	Foods Included	N (%) Ever in last 7 days	Mean (SD) days in last 7 days	N (%) Ever in last 7 days	Mean (SD) days in last 7 days
Cereal, roots &tubers	Millet, rice, sorghum, maize, cassava	949(99.0)^*^	6.7(±1.0)	1415(99.8)	6.7(±0.9)
Legumes	Cowpea, pea, lentils, peanuts, other nuts	563(58.7)^**^	3.4(± 2.0)^*^	1019(71.9)	3.7(±2.0)
Dairy	Milk, yogurt, cheese, other dairy products	286(29.8)	3.7(±2.2)	373(26.3)	3.8(2.3)
Flesh foods	Meat, poultry, fish	427(44.5)^**^	2.5(±1.6)	772(54.4)	2.6(±1.7)
Eggs	Eggs	203(21.2)	2.4(±1.6)	269(19.0)	2.1(±1.4)
Vitamin A rich fruits & vegetables	Pumpkin, carrots, sweet potato, dark green leaves, mango or papaya ripe	897(93.5)^*^	6.3(±2.6)	1356(95.6)	6.2(±2.3)
Other fruits &vegetables	Other vegetables (tomato, onion, cucumber, zucchini, eggplant, cabbage, okra), other fruits (banana, guava, wate0rmelon)	449(46.8)^**^	4.1(±2.3)^*^	792(55.9)	4.4(±2.2)
Fats &oils	Oil, butterfat, coconut milk, butter	600(62.6)^**^	4.4(±2.2)	1058(74.6)	4.3(±2.1)
Dietary diversity score	Sum of 8 food groups		2.7 (1.1)^*^		2.9 (1.0)

^*^Significant at 0.05 level; ^**^significant at< 0.001

Counts and means evaluated for significant difference between groups using chi-square and t-test, respectively. Consumption of food groups is calculated among children reported to consume the food group

There was no significant association between individual food group intakes or overall DDS with treatment recovery at program discharge or 12 weeks (Table 3). However, the consumption of eggs was protective against death while consumption of vitamin A rich fruits and vegetables was associated with greater risk of mortality. For every additional day of egg consumption, the risk of death decreased by 47% (RR: 0.53, 95% CI: 0.39–0.70) at program discharge and 34% (RR: 0.66, 95% CI: 0.45–0.96) at 12 weeks of follow-up. Each additional day of vitamin A rich fruits and vegetables consumption was associated with a 30% increase in risk of death at program discharge (RR: 1.30, 95% CI: 1.08–1.56) and a 19% (RR: 1.19, 95% CI: 1.03–1.36) increase in risk at 12 weeks. There was no significant association between individual food group intakes with transfer to hospital.

**Table 3:** Association of dietary intake and DDS with outcomes at discharge and 12 weeks

Food group	Program discharge^a^		12 weeks^a^	
	RR Unadjusted (95% CI)	RR adjusted (95% CI)	RR Unadjusted (95% CI)	RR adjusted (95% CI)
Treatment recovery				
Cereals, roots & tubers	1.02(0.97, 1.08)	1.00(0.96, 1.05)	1.01(0.95, 1.07)	0.98(0.94, 1.03)
Flesh foods	1.00(0.96, 1.04)	1.00(0.97, 1.03)	1.00(0.96, 1.05)	1.00(0.97, 1.03)
Legumes	1.01(0.98, 1.04)	1.01(0.99, 1.03)	1.01(0.98, 1.04)	1.01(0.98, 1.03)
Dairy	1.02(0.98, 1.06)	1.00(0.96, 1.04)	1.01(0.97, 1.06)	0.99(0.94, 1.04)
Eggs	0.98(0.91, 1.06)	0.98(0.93, 1.04)	0.98(0.91, 1.07)	0.98(0.92, 1.04)
Vitamin A rich fruits & vegetables	1.01(0.99, 1.03)	1.01(0.99, 1.02)	1.01(0.99, 1.03)	1.01(0.99, 1.02)
Other fruits & vegetables	1.00(0.97, 1.03)	0.99(0.97, 1.01)	1.00(0.96, 1.02)	0.98(0.95, 1.00)
Fat and oils	1.01(0.98, 1.04)	1.01(0.99, 1.03)	1.01(0.98, 1.04)	1.00(0.98, 1.03)
Diet diversity score	1.06(1.03, 1.09)**	1.02(0.98, 1.06)	1.04(1.02, 1.08)*	0.96(0.91, 1.02)
Death				
Cereals, roots & tubers	1.08(0.56, 2.10)	1.43(0.69, 2.99)	0.98(0.71, 1.35)	0.99(0.71, 1.39)
Flesh foods	1.15(0.81, 1.63)	1.16(0.88, 1.53)	0.96(0.72, 1.28)	0.96(0.67, 1.36)
Legumes	0.94(0.69, 1.28)	0.97(0.80, 1.18)	0.97(0.79, 1.20)	1.01(0.83, 1.24)
Dairy	0.59(0.28, 1.26)	0.74(0.39, 1.39)	0.91(0.71, 1.18)	0.70 (0.45, 1.10)
Eggs	0.60(0.24, 1.48)	0.53(0.39, 0.70)**	0.65(0.40, 1.06)	0.66(0.45, 0.96)*
Vitamin A rich fruits & vegetables	0.91(0.72, 1.14)	1.30(1.08, 1.56)*	0.91(0.79, 1.04)	1.19(1.03, 1.36)*
Other fruits & vegetables	0.94(0.68, 1.29)	1.01(0.67, 1.53)	1.05(0.85, 1.30)	1.06(0.79, 1.43)
Fat and oils	0.72(0.51, 1.03)	0.75(0.51, 1.10)	0.98(0.82, 1.17)	0.87(0.68, 1.13)
Diet diversity score	1.17(0.76, 1.82)	1.54(0.93, 2.55)	0.86 (0.61, 1.21)	0.80(0.54, 1.20)
Transfer to hospital				
Cereals, roots & tubers	0.93(0.87, 1.00)	0.97(0.91, 1.03)	0.98(0.91, 1.05)	1.02(0.95, 1.10)
Flesh food	1.01(0.94, 1.08)	0.99(0.92, 1.06)	1.00(0.94, 1.07)	1.00(0.94, 1.06)
Legumes	0.96(0.91, 1.01)	0.96(0.91, 1.02)	0,97(0.92, 1.03)	0.98(0.93, 1.03)
Dairy	0.96(0.90, 1.02)	0.99(0.92, 1.06)	0.97(0.92, 1.03)	1.01(0.94, 1.07)
Eggs	1.01(0.90, 1.13)	1.00(0.90, 1.10)	1.00(0.90, 1.12)	1.09(0.97, 1.23)
Vitamin A rich fruits & vegetables	0.98(0.95, 1.02)	0.99(0.96, 1.03)	1.00(0.97, 1.03)	1.01(0.98, 1.04)
Other fruits & vegetables	0.99(0.94, 1.04)	1.03(0.98, 1.08)	1.01(0.96, 1.05)	1.04(1.00, 1.09)
Fat and oils	0.99(0.94, 1.04)	1.01(0.96, 1.05)	0.99(0.94, 1.03)	1.01(0.97, 1.05)
Diet diversity score	0.89(0.83, 0.95)	0.94(0.86, 1.02)	0.95(0.89, 1.01)	1.02(0.96, 1.09)

*Abbreviations*: *CI* confidence interval, *RR* relative risk, *significant at 0.05 level; **significant at < 0.001

^a^Analysis excludes 22 children that were exclusively breastfed as they were not expected to consume complementary foods. All food groups were modeled as continuous variables. Covariate selection based on significance in univariate models at p < 0.20. RR for individual food groups reflects association for a 1-day unit increase in consumption and RR for DDS reflects association for a 1 unit increase in DDS

There was no significant association of DDS with the rate of weight change among recovered children. However, each additional day of legume and nut consumption was associated with increased rate of weight gain at discharge (mean change 0.16 g/kg/day, 95% CI: 0.03–0.30) and an additional day of vitamin A rich fruits and vegetable consumption with a small increase in weight gain at 12 weeks (mean change 0.06 g/kg/day, 95% CI: 0.01–0.11) (Table 4).

**Table 4:** Food groups consumed by rate of weight and height gain among recovered children (*N* = 1538)

	Mean weight gain (g/kg/day)								Mean height gain (mm/day)	
	1 week		2 week		Discharge		12 weeks		12 weeks	
Food group	Mean	Adj. (95% CI)	Mean	Adj. (95% CI)	Mean	Adj. (95% CI)	Mean	Adj. (95% CI)	Mean	Adj. (95% CI)
Cereals, roots and tubers	9.38	−0.07 (−0.47, 0.33)	6.51	− 0.05 (− 0.26, 0.17)	4.32	0.05 (− 0.09, 0.19)	2.01	− 0.04 (− 0.10, 0.02)	4.96	0.10 (− 0.12, 0.32)
Meats	9.56	− 0.13 (− 0.43, 0.17)	4.81	0.15 (− 0.07,0.37)	4.62	0.03 (− 0.08, 0.14)	1.82	0.04 (− 0.01, 0.08)	6.86	− 0.29 (− 0.29, 0.04)
Legumes	8.20	0.02 (− 0.18, 0.22)	5.89	− 0.07 (− 0.22, 0.08)	3.61	0.16 (0.03, 0.30)^*^	1.73	0.02 (− 0.01, 0.05)	5.42	− 0.05 (− 0.17, 0.07)
Dairy	11.56	− 0.17 (− 0.51, 0.17)	6.58	−0.13 (− 0.29, 0.04)	4.88	−0.02 (− 0.14, 0.08)	1.65	−0.03 (− 0.08, 0.01)	6.36	−0.31 (− 0.65, 0.03)
Eggs	7.28	−0.02 (− 0.57, 0.54)	4.55	−0.13 (− 0.41, 0.16)	4.01	0.07 (− 0.13, 0.26)	1.43	0.00 (− 0.09, 0.09)	4.17	−0.15 (− 0.45, 0.15)
Vitamin A rich fruits and vegetables	8.67	0.02 (−0.13, 0.18)	5.99	0.00 (−0.08, 0.09)	4.40	0.03 (−0.03, 0.08)	1.45	0.06 (0.01, 0.11)^*^	5.61	0.05 (−0.04, 0.13)
Other fruits and vegetables	7.14	−0.05 (− 0.27, 0.17)	4.72	− 0.06 (− 0.18, 0.05)	3.87	−0.00 (− 0.08, 0.07)	1.71	0.03 (− 0.05, 0.10)	5.58	−0.03 (− 0.15, 0.09)
Fat and oils	8.54	−0.12 (− 0.32, 0.08)	5.53	−0.03 (− 0.13, 0.07)	4.21	−0.02 (− 0.09, 0.05)	1.60	0.01 (− 0.02, 0.04)	6.18	−0.05 (− 0.16, 0.05)
Diet diversity score	8.41	0.15 (−0.21, 0.51)	6.52	−0.13 (− 0.40, 0.14)	4.31	0.12 (− 0.01, 0.25)	1.64	0.03 (− 0.02, 0.09)	5.59	0.03 (− 0.17, 0.23)

Abbreviations: *CI* confidence interval, *Adj* Adjusted models; ^*^Significant at 0.05 level

Analysis excludes 22 children that were exclusively breastfed they were not expected to consume complementary foods. Analysis modeled using linear regression

Exploratory analyses included an assessment of any non-linear relationship between the exposures of interest (food group intake and DDS) and all outcomes using restricted cubic splines, as well as alternative categorizations of dietary diversity (e.g. binary [±4 food groups/day], defining intake as a minimum of ≥3 times in the last 7 days and using the WHO dietary diversity index for infant and young feeding [19]); all results were similar to those presented here (results not shown).

Table 5 shows predictors of nutritional recovery. The severity of WHZ at admission was marginally associated with the likelihood of recovery, with children with WHZ < − 3 having lower odds of recovery compared to better nourished children (OR: 0.21, 95% CI: 0.04–1.03, *p* = 0.05). Further, children recruited into the study in the lean months, were less likely to recover compared children treated in the non-lean season (OR: 0.51, 95% CI: 0.26–1.01, p = 0.05). Additionally, seeking health care in the last 30 days was associated with increased risk of recovery (OR: 2.29, 95% CI: 1.1–4.47, *p* = 0.01).

**Table 5:** Predictors of nutritional recovery among 2377 children treated for SAM in Niger

Variable	Univariate OR (95% CI)	Adjusted OR (95% CI)
Child age		
6-11 months	0.51(0.41, 0.65)	0.93(0.24, 3.58)
12–23 months	0.77(0.61, 0.98)	1.68(0.54, 5.17)
Wasting		
Moderate wasting (− 3.0 < =WHZ < − 2.0)	0.65(0.41, 1.01)	0.40(0.08, 2.01)
Severe wasting (WHZ < − 3.0)	0.41(0.26, 0.63)	0.21(0.04, 1.03)
Number of children under 5 years	1.05(0.98, 1.12)	1.23(0.92, 1.64)
Maternal age	1.02(1.01, 1.04)	1.03(0.92, 1.08)
Lean season at time of enrolment	0.59(0.50, 0.70)	0.51(0.26, 1.01)
Household size	1.15(0.95, 1.40)	0.76(0.34, 1.72)
Sought health care for child in the last 30 days	1.57(1.07, 2.30)	2.29(1.18, 4.47)
Positive rapid malaria test	1.45(1.23, 1.72)	1.89(0.93, 3.84)
Anti-malarial medication received in previous 7 days	1.44(0.98, 2.12)	1.37(0.71, 2.62)
Antibiotic use prior to study	0.66(0.37, 1.17)	0.55(0.17, 1.78)
Runny nose	1.74(1.40, 2.15)	0.53(0.22, 1.28)
Respiratory rate	0.98(0.97, 1.00)	0.96(0.88, 1.05)
Cough	1.40(1.11, 1.77)	1.10(0.36, 3.36)
Child anemia (hemoglobin < 8.5 g/dL)	1.46(1.21, 1.77)	1.18(0.54, 2.60)
Currently breastfeeding	0.66(0.55, 0.79)	0.74(0.29, 1.86)
Drug regimen (Amoxicillin)	1.15(0.97, 1.36)	0.82(0.45, 1.51)
Male child	0.82(0.69, 0.97)	1.22(0.63, 2.35)
Consulting for child’s health	1.32(1.07, 1.63)	–
Child received Ready to use supplementary foods(RUSF) prior to study	1.42(1.06, 1.90)	1.17(0.41, 3.35)

Abbreviations: *CI* confidence interval, *OR* odds ratio

Analysis excludes 22 children that were exclusively breastfed. They were not expected to consume complementary foods. Analysis modeled using logistic regression

## Discussion

We evaluated the relationship of dietary intake and diversity with nutritional program outcomes and response to treatment among children with uncomplicated SAM in Niger. Overall, we found that children with more diverse diets at admission did not have superior rates of nutritional recovery, decreased occurrences of hospitalization or death, nor increased rates of weight and height gain. The consumption of eggs was protective against death at program discharge and at 12 weeks of follow-up. The consumption of vitamin A rich fruits and vegetables however increased risk of mortality in children treated for uncomplicated SAM in Niger.

Poor dietary diversity has previously been associated with stunting and edema [20, 21], but to our knowledge, there has been no previous study of its potential role in SAM treatment. Our findings provide no evidence to support a role for diet diversity in SAM treatment, suggesting that other factors may more strongly contribute to recovery. Several possibilities may explain the observed lack of association in this population. During treatment, children are provided with sufficient ready to use therapeutic food (RUTF) to meet nutritional needs during rehabilitation, and adherence to prescribed RUTF may be more influential in recovery, compared to dietary diversity at admission. Previous work by Yebyo et al. found that Ethiopian children who consumed one additional sachet of RUTF increased the likelihood of recovery by 4% (95% CI:  1.03, 1.05, *p* < 0.001) [9]. Our study collected information on reported RUTF consumption during treatment, and reported compliance was not significantly associated with recovery (results not shown).

We considered that dietary diversity may be important for treatment response through the consumption of specific food groups, such as animal-source foods previously shown to be important for improving diet quality, micronutrient status, and growth [20, 22]. Our study did not find evidence that consumption of animal-source foods, such as meats and dairy, influenced nutrition recovery or rate of weight gain, but intake of dairy and meats was low in this population. Reported diets may not have included sufficient quantities or variability of food group intake to influence recovery. Egg consumption, however, was associated with a significant reduction in the risk of death at discharge and 12 weeks. This finding is particularly important given limited consumption of eggs in the study. Eggs contain almost complete protein, B vitamins, and essential fatty acids (EFA) including the omega 6 fatty acid, arachidonic acid, and omega 3 fatty acid, docosahexanoic acid [23, 24]. EFA have been associated with increased growth and development in children [23, 24]. Ionnotti et al. further suggest that since eggs contain critical micro- and macronutrients in highly bioavailable forms, they may have value in enhancing plant-based diets for children in developing countries [23, 24]. Mosites et al. also provide evidence for the role of EFA and complete protein from egg consumption to protect against mortality in Western Kenya [25]. An unexpected finding from this study was that the consumption of vitamin A rich fruits and vegetables was associated with an increased risk of death at discharge and 12 weeks. A potential explanation for this finding is that in poor resource areas, consumption of vitamin A rich vegetables could signify the unavailability of more nutrient dense, and more expensive, animal-source foods.

We sought to determine independent predictors of treatment recovery aside from dietary intake and diversity. We found that WHZ severity had a trend towards independently decreasing the odds of recovery. This finding was in line with Gebremichael et al., who showed that weight at recruitment lower than the mean increased nutritional recovery time for severely malnourished children [26]. James et al. also found that WHZ less than − 3.5 was associated with lower odds for recovery among children in Myanmar [27]. Similarly, in an inpatient program of non-edematous children aged 6–59 months in Uganda, low WHZ at admission was a significant risk factor for mortality [28]. This association is supported by the mechanism in which acute malnutrition impairs a child’s ability to respond to infection and environmental stressors [29]. We also found a marginal association with season (*P* = 0.05), as children recruited in the lean months (May to August) were less likely to recover compared children treated in the non-lean season. Although RUTF is provided in quantities sufficient to meet nutrient needs for recovery, lower food availability during the lean season may increase sharing within the household or sales of RUTF intended for the index child [30]. Season may therefore be predictive of recovery. Finally we also found that seeking health care in the last 30 days was associated with increased recovery. This is plausible given the interaction between malnutrition and infection [31] . Recent consultation at a health service may also be a proxy for positive health care and dietary behaviors. A study in Malawi found poorer nutritional recovery in younger children, those with marasmic kwashiorkor, stunting, infection, and cough at enrolment [32]. Another program in Ethiopia found that diarrhea, vomiting, average weight gain, amoxicillin and de-worming medication were positively associated with recovery [9]. Differences in predictors of nutritional recovery may be due to the contextual nature of malnutrition and SAM, with the relative importance of determinants differing based on the local context.

This study has several strengths and limitations. To our knowledge, this study provides the first evidence related to the possible role of dietary intake and diversity in the nutritional recovery of severely malnourished children. Other strengths of the study include a large study population, and an in-depth assessment of clinical and socio-economic factors associated with SAM in this setting. A key limitation of the study is that dietary intake for children was measured at baseline only, prior to the start of treatment.

Further, we could not apply a minimum amount of food intake in the DDS calculation. Studies have shown that when minimum amounts of consumption are considered (eg at least 10 g or 15 g of food consumed for it contribute to food groups), the association of DDS with nutrient adequacy and sensitivity of the DDS indicator are improvexd [10, 17, 33]. Incorporation of portion sizes may help DDS identify children with low dietary diversity, [33] although consideration of a cut-off of consumption of food groups at least 3 times did not substantially change the results (data not shown).

## Conclusion

In this study, neither dietary intake nor diversity were associated with nutrition recovery or response to treatment of children with uncomplicated SAM. It is feasible that beyond dietary diversity, the consumption of energy dense foods like eggs may be more important for recovery from SAM. There is need for continued research to further elucidate the drivers of nutritional recovery from acute malnutrition in different settings to inform recommendations to support nutritional recovery and response to treatment.

## Abbreviations

CI: Confidence interval
DDS: Diet diversity score
EFA: Essential fatty acids
FFQ: Food frequency questionnaire
MSF: Médecins Sans Frontières
MUAC: Mid-upper arm circumference
OR: Odds ratio
RR: Relative risk
RUTF: Ready to use therapeutic food
SAM: Severe acute malnutrition
WHO: World Health Organization
WHZ: Weight-for-height Z score
